# Development of an Interpretable QSAR Model for Predicting Coagulation Factor XIIa Inhibitors Using Ensemble Machine Learning

**DOI:** 10.3390/ph19091426

**Published:** 2026-09-09

**Authors:** Ali Onur Kaya, Mert Can Emre

**Affiliations:** 1 Radiotherapy Department, Health Services Vocational School, Akdeniz University, 07070 Antalya, Türkiye; 2 Motor Vehicles Department, Sorgun Vocational School, Yozgat Bozok University, 66000 Yozgat, Türkiye; m.can.emre@yobu.edu.tr

**Keywords:** Coagulation factor XIIa, FXIIa inhibitors, quantitative structure–activity relationship (QSAR), machine learning, anticoagulant drug discovery

## Abstract

**Background/Objective:** Activated coagulation factor XII (FXIIa) is a component of the contact activation pathway and a pharmacologically relevant target in contact-system-associated processes. In this study, scaffold-aware and interpretable machine-learning QSAR models were developed for human FXIIa activity. **Methods:** Bioactivity records for the human single protein target CHEMBL2821 were retrieved from ChEMBL release 37. Modeling was restricted to exact IC50 measurements from assays explicitly referring to FXIIa, Factor XIIa, or activated Factor XII. Median-consolidated pIC50 values and two-dimensional Mordred descriptors were evaluated using leakage-safe preprocessing, scaffold-disjoint validation, Y-randomization, applicability domain analysis, structural similarity auditing, and SHAP interpretation. **Results:** The regression dataset comprised 424 compounds and 166 Bemis–Murcko scaffolds in this study. The Gradient Boosting regressor achieved R^2^ = 0.7560, RMSE = 0.6924, and MAE = 0.4965 on the locked scaffold-disjoint test set (*n* = 85); across 50 repeated scaffold partitions, the mean R^2^ was 0.6892 ± 0.1515. The classification model achieved ROC-AUC = 0.9453, PR-AUC = 0.9807, balanced accuracy = 0.7561, and MCC = 0.5972 (*n* = 73). Y-randomization supported nonrandom predictive signals (empirical *p* = 0.0099). **Conclusions:** The models support computational prioritization within the represented FXIIa chemical domain, while prospective evaluation of independently generated compounds remains necessary.

## 1. Introduction

Thrombotic disorders remain major causes of morbidity and mortality, and conventional anticoagulation is inherently associated with bleeding risk because many established targets are required for normal hemostasis. Consequently, increasing attention has been focused on coagulation factors that contribute to pathological thrombosis while playing a less critical role in physiological hemostasis. Activated coagulation factor XII (FXIIa) has emerged as a pharmacologically attractive target in this context [1,2,3,4].

Factor XII (FXII) is a plasma serine protease zymogen and a central component of the contact activation system. Following activation, FXIIa promotes factor XI activation and participates in the plasma kallikrein–kinin system [2,3,5,6,7,8]. Through these interconnected pathways, FXII/FXIIa contributes to coagulation, bradykinin generation, vascular responses, and inflammatory signaling [3,8,9]. Experimental studies have shown that genetic or pharmacological interference with FXII/FXIIa can attenuate pathological thrombus formation without producing a bleeding phenotype associated with the inhibition of essential hemostatic factors [4,10,11,12,13,14]. The contact system also participates in inflammatory, host response, and bradykinin-mediated processes [6,7,8,9,15,16,17], emphasizing its broader and context-dependent biological role.

Clinical evidence has established FXIIa as a pharmacologically tractable therapeutic target. In the phase III VANGUARD trial, the FXIIa-targeting monoclonal antibody garadacimab reduced the number of hereditary angioedema attacks [18]. Because hereditary angioedema is primarily a bradykinin-mediated disorder, these findings should not be interpreted as direct clinical evidence of the antithrombotic efficacy of icatibant [19]. Nevertheless, preclinical FXII/FXIIa-directed interventions, including inhibitory antibodies and Infestin-4, have demonstrated antithrombotic effects in experimental systems [20,21,22].

These findings have encouraged the development of FXIIa-targeting agents, including small-molecule inhibitors of FXIIa. Medicinal chemistry studies and reviews have described structurally diverse compounds and opportunities for improving potency, selectivity, covalent engagement, and pharmacological properties [23,24,25,26,27,28]. Such experimentally characterized chemical series provide an appropriate basis for ligand-based computational modeling studies.

Nevertheless, the construction of an FXIIa QSAR dataset presents important challenges. Public bioactivity repositories integrate measurements from different assay conditions, formats, and endpoint definitions, whereas assignment to a common database target does not necessarily establish an equivalent experimental target state. This is particularly relevant because ChEML target CHEMBL2821 is formally annotated as coagulation factor XII, whereas the present study concerns activity measured against FXIIa.

Endpoint selection introduces additional heterogeneity in the study. Both IC50 and Ki describe inhibitory activity but are not experimentally interchangeable, and IC50 is dependent on assay conditions [29,30,31]. ChEMBL provides structured bioactivity and assay information [32]; however, careful dataset definition and chemical structure curation remain essential for QSAR modeling [33,34]. Therefore, the present study adopted an IC50-specific and assay-context-specific curation strategy rather than pooling non-equivalent potency endpoints.

The predictive QSAR performance depends strongly on the dataset construction and validation strategy [35,36,37,38]. Random compound-level splitting may distribute closely related analogs between the development and test sets, potentially producing optimistic estimates of generalization, whereas alternative partitioning strategies can materially affect apparent prospective performance [39,40]. Scaffold-aware validation addresses this problem by separating compounds according to core structural frameworks, which are defined using Bemis–Murcko scaffolds [41]. Because performance can still depend on the particular scaffolds withheld, repeated scaffold-disjoint assessment provides useful complementary information beyond a single locked partition.

Data-dependent preprocessing must also remain confined to the training data to avoid information leakage. Y-randomization provides a negative control for non-random predictive signals [35,36,37,38], whereas applicability domain analysis evaluates whether compounds are represented within the modeled molecular space [42,43,44]. These approaches address different aspects of model validation and should not be interpreted as guarantees of the accuracy of individual predictions.

Binary classification introduces additional dependence on operational activity thresholds, and scaffold separation does not necessarily eliminate residual molecular similarities. Threshold sensitivity and structural similarity analyses are therefore useful complements to scaffold-disjoint evaluation. Model interpretation also requires caution. SHapley Additive exPlanations (SHAP) quantify feature contributions [45,46], but correlated molecular descriptors may distribute attribution across related variables. Therefore, highly ranked descriptors should be interpreted as model-influential features rather than causal determinants of FXIIa activity.

Previously ligand-based modeling has demonstrated the feasibility of QSAR approaches for FXIIa. Chen and Visco developed PCA-GA-SVM models for virtual screening of FXIIa inhibitors [47], while our previous machine-learning studies investigated related coagulation targets, including Factor X and FXIa [48,49]. Direct numerical comparisons are inappropriate because these studies differ in datasets, endpoints, chemical distributions, and validation strategies.

Accordingly, this study developed scaffold-aware and interpretable machine-learning QSAR models for human FXIIa activity using an IC50-specific, assay-context-defined ChEMBL dataset. The workflow combined model-independent scaffold partitioning, leakage-safe preprocessing, repeated scaffold-disjoint assessment, Y-randomization, complementary applicability domain analyses, classification-threshold sensitivity, structural-similarity auditing, and SHAP stability analysis to characterize the predictive performance and its limitations for computational compound prioritization.

## 2. Results

### 2.1. Dataset Curation and Definition of the FXIIa Modeling Domain

Reconstruction of the CHEMBL2821 bioactivity space from ChEMBL release 37 identified 3016 activity records associated with the human single-protein target, formally annotated as coagulation factor XII. Restricting the search to IC50 measurements yielded 1646 records. Sequential application of the exact-relation, nanomolar-unit, positive-value, activity-validity, and structure-quality criteria yielded 595 eligible activity records corresponding to 547 standardized compounds before assay-context restrictions.

**Figure 1:**
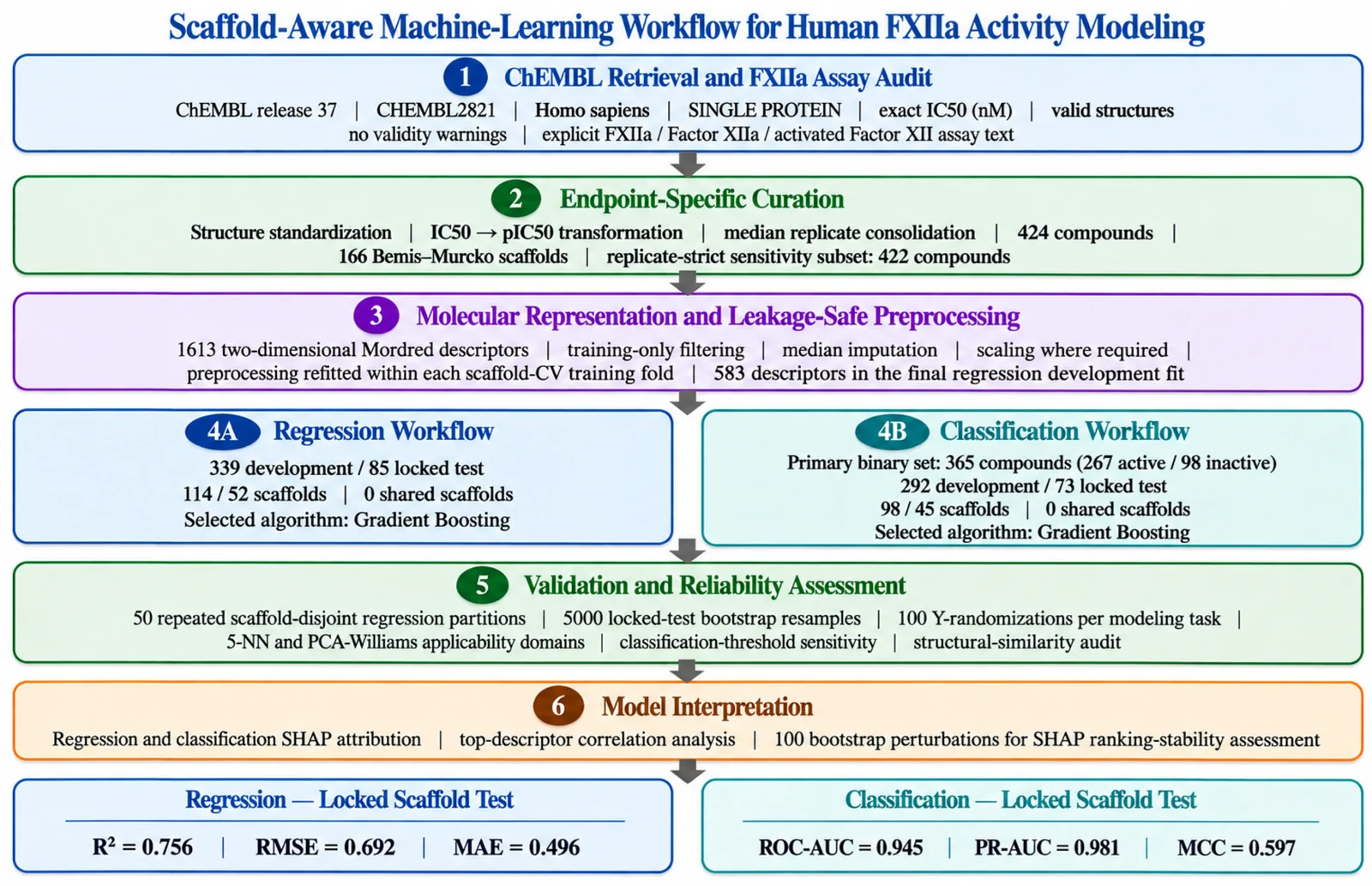
Overview of the assay-context-specific, scaffold-aware machine-learning workflow for human FXIIa activity modeling.

Because the present study specifically addressed the activated protease FXIIa, assay descriptions were subsequently audited for explicit reference to “FXIIa,” “Factor XIIa,” or “activated Factor XII.” This restriction defined the primary modeling domain and yielded 424 compounds representing 166 Bemis–Murcko scaffolds [41]. The resulting pIC50 values ranged from 2.5596 to 10.0000, with a mean of 6.9617, median of 7.3111, and standard deviation of 1.3713 (Table 1; Figure 1). Compound-level curation information is provided in the Supplementary Data Sheet S1.

Replicate variability was examined separately from the primary dataset. In the broader 547-compound IC50 audit population, the four replicate groups exhibited a within-compound pIC50 range greater than 1.0 log unit. Following the restriction to the explicit FXIIa assay domain, two of these groups remained in the 424-compound primary population. Since this level of disagreement was not considered a universal exclusion criterion across heterogeneous biochemical assays, both compounds were retained in the primary dataset. Their exclusion generated a 422-compound replicate-strict-sensitivity population. Complete replicate-level auditing is provided in Supplementary Data Sheet S2.

Thus, no compounds were removed from the primary modeling population solely because of replicate dispersion or predefined physicochemical or lead-like criteria.

### 2.2. Definition of the Binary Classification Space and Threshold Sensitivity

For the primary classification task, compounds with pIC50 ≥ 7.0 were defined as active, whereas compounds with pIC50 < 6.0 were defined as inactive. The 59 compounds with 6.0 ≤ pIC50 < 7.0 were treated as an intermediate gray zone and excluded from the primary binary model development. This resulted in a classification population of 365 compounds, comprising 267 active and 98 inactive compounds. Compound-level class assignments are provided in Supplementary Data Sheet S3.

Because these boundaries were operational modeling definitions rather than universal biological thresholds, the classification performance was additionally examined under four alternative activity definitions. To distinguish the effect of changing the activity definition from that of generating a different scaffold partition, the scaffold allocation frozen for the primary classification task was preserved across all threshold-sensitivity analyses.

Compounds newly entering an alternative scenario from the original gray zone were included in the performance assessment only when their Bemis–Murcko scaffold had a development/test assignment in the frozen primary partition. Consequently, the number satisfying the activity definition could exceed the number entering the frozen-partition analysis. The active ≥ 6.5/inactive < 6.0 scenario contained 405 threshold-eligible compounds, of which 389 were analyzed; the active ≥ 7.0/inactive < 6.5 scenario contained 384 eligible and 376 analyzed compounds; and the single 6.5 cutoff contained 424 eligible and 400 analyzed compounds. All 345 compounds eligible under the expanded 5.5–7.0 Gy zone definition retained a frozen scaffold assignment.

Across these definitions, the locked-test ROC-AUC ranged from 0.9223 to 0.9594, whereas the balanced accuracy and MCC were more sensitive to the selected boundaries (Table 2). The active ≥ 7.0/inactive < 6.5 definition produced the highest ROC-AUC (0.9594) and balanced accuracy (0.8061), whereas the expanded 5.5–7.0 gray-zone definition yielded the highest MCC (0.6782). Nevertheless, the original pIC50 < 6.0 versus pIC50 ≥ 7.0 definition was retained as the primary classification task because it was prespecified independently of the locked-test performance and maintained a one-log-unit separation between the modeled classes. The complete threshold definitions and performance results are provided in Supplementary Data Sheet S4.

### 2.3. Scaffold-Disjoint Regression Partition and Leakage-Safe Descriptor Processing

**Figure 2:**
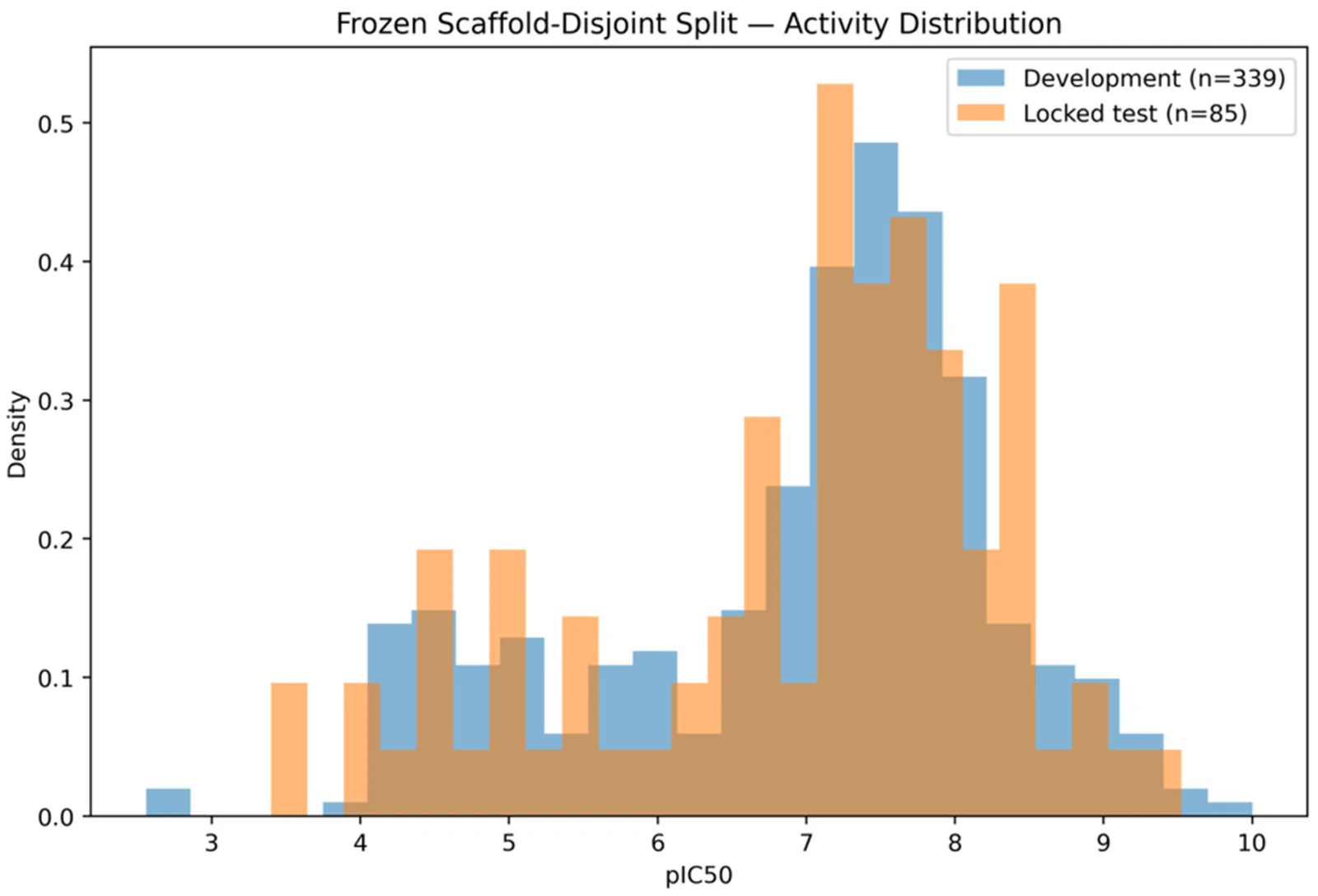
pIC50 distributions of the development and locked scaffold-disjoint regression test sets.

The 424-compound regression population was partitioned using Bemis–Murcko scaffolds [43] as indivisible, structural groups. Among the 2000 model-independent candidate scaffold-disjoint partitions, the selected frozen partition, candidate 1952 (seed 20,262,779), contained 339 development compounds representing 114 scaffolds and 85 locked-test compounds representing 52 scaffolds, with no shared scaffolds between the two populations (Table 3; Figure 2). The compound-level regression partition assignments are provided in Supplementary Data Sheet S5.

The mean pIC50 values were 6.9666 and 6.9423 for the development and locked-test populations, respectively, whereas the corresponding medians were 7.3188 and 7.3002. The two activity distributions showed close agreement in a two-sample Kolmogorov–Smirnov comparison (*p* = 0.9922; Figure 2).

Descriptor generation using the Mordred molecular descriptor framework [44] produced 1613 initial two-dimensional descriptors, of which 94 were entirely missing across the complete molecular matrix. Importantly, data-dependent feature filtering was performed only after freezing the scaffold partition. Using the 339-compound development population, 155 descriptors exceeding 20% missingness were removed, followed by 195 descriptors with variance ≤ 1 × 10^−12^, and 680 descriptors were removed during redundancy filtering at |r| > 0.95. The resulting frozen-development-derived representation contained 583 descriptors.

When preprocessing was independently refitted within the five scaffold-cross-validation training folds, 591, 589, 580, 570, and 572 descriptors were retained. These fold-specific differences confirm that feature filtering was performed independently within each training fold rather than being defined globally using the validation or locked-test information.

### 2.4. Regression Algorithm Benchmarking and Final Model Selection

Nine regression algorithms were evaluated using five-fold scaffold-grouped cross-validation. Initial benchmarking identified Extra Trees, XGBoost, and Gradient Boosting as the three strongest candidates (Table 4). The complete benchmark results are provided in Supplementary Data Sheet S6.

Extra Trees achieved an initial mean scaffold-CV R^2^ of 0.4453 and a pooled OOF R^2^ of 0.6054. XGBoost produced corresponding values of 0.4409 and 0.5863, whereas Gradient Boosting yielded 0.4286 and 0.5843.

The three leading algorithms were subsequently evaluated using 12 predefined configurations for each algorithm. Among the resulting 36 configurations and 180 fold-level fits, Gradient Boosting provided the strongest mean scaffold-CV performance. The selected configuration achieved a mean CV R^2^ = 0.4909 ± 0.3493, with pooled OOF R^2^ = 0.6130, RMSE = 0.8470, and MAE = 0.6439.

The final model used 300 estimators, a learning rate of 0.03, maximum depth of 3, minimum sample split of 4, minimum sample leaf of 3, subsample of 0.9, unrestricted maximum features, and Huber loss. Model selection was completed using the development set results before evaluating the locked-test population. The full configuration-selection results are provided in Supplementary Data Sheet S7.

The difference between the mean fold-wise R^2^ and pooled OOF R^2^ reflects the heterogeneity in fold size, response distribution, and scaffold-specific prediction difficulty. Model selection was based entirely on a predefined development set scaffold validation framework.

### 2.5. Locked and Repeated Scaffold-Disjoint Regression Performance

Following completion of model selection, the frozen Gradient Boosting pipeline was fitted to the complete development population and evaluated on the previously untouched 85-compound locked scaffold-disjoint test set.

**Figure 3:**
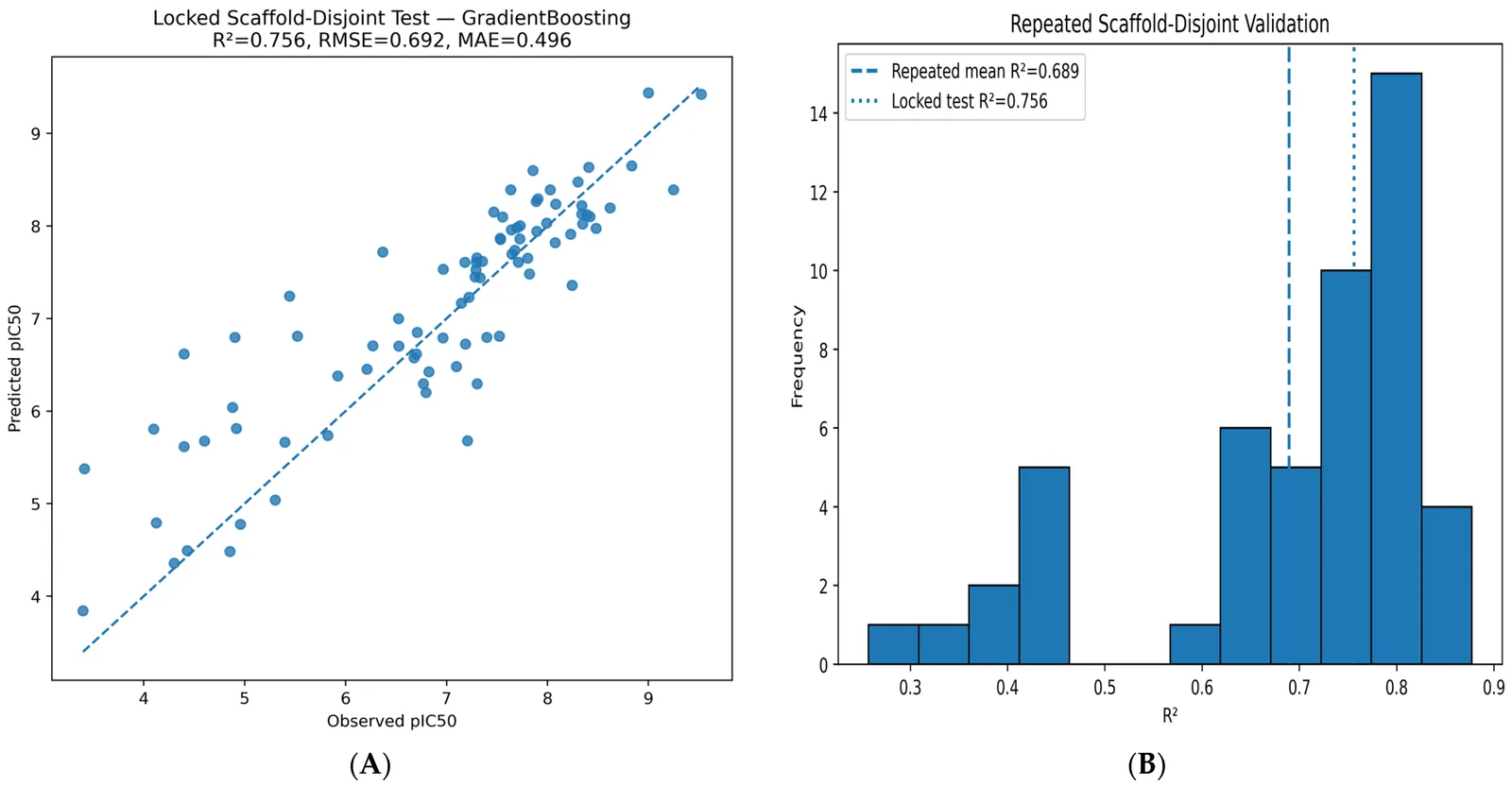
Regression performance of the final Gradient Boosting model. (**A**) Observed versus predicted pIC50 values for the locked scaffold-disjoint test set. (**B**) Distribution of R^2^ values across 50 repeated scaffold-disjoint partitions.

The model achieved R^2^ = 0.7560, RMSE = 0.6924, and MAE = 0.4965 on the locked test set (Table 5; Figure 3A). Compound-level observed and predicted values are provided in Supplementary Data Sheet S8.

Nonparametric bootstrap assessment using 5000 resamples yielded an R^2^ median of 0.7546 and an empirical 95% percentile interval of 0.6454–0.8398. The corresponding intervals were 0.5448–0.8289 for RMSE and 0.3986–0.6032 for MAE, respectively.

Because the performance of a single scaffold partition can depend on the chemical series assigned to the test population, the validation was extended to 50 repeated scaffold-disjoint partitions (Figure 3B). Across these repetitions, the mean R^2^ was 0.6892 ± 0.1515, with a median of 0.7431. The observed R^2^ values ranged from 0.2568 to 0.8774, and the empirical 2.5th–97.5th percentile range was 0.3571–0.8501. The mean RMSE and MAE were 0.6959 ± 0.1426 and 0.5279 ± 0.1224, respectively. Complete repeated-partition results are provided in Supplementary Data Sheet S9.

These results indicate an appreciable predictive signal across scaffold-separated evaluations while also demonstrating meaningful partition dependence. Accordingly, the locked-test R^2^ of 0.7560 was interpreted together with the repeated scaffold-disjoint distribution rather than as an isolated performance estimate.

For the repeated scaffold partitions, the empirical 2.5th–97.5th percentile range describes the variation among alternative scaffold allocations and is not interpreted as a confidence interval.

### 2.6. Regression Y-Randomization

The possibility that the regression performance arose from chance associations between the molecular descriptors and activity was investigated using 100 Y-randomization experiments. The observed scaffold-OOF R^2^ of 0.6130 was clearly separated from the random distribution. The permuted-response models produced a mean R^2^ of −0.2157 ± 0.0688, a median of −0.2092, and a maximum of −0.0964. None of the 100 randomized models reached or exceeded the observed scaffold OOF performance (Table 6).

Using the predefined empirical calculation

(1) $$p_{emp}=\frac{1+\sum_{i=1}^{N}I\left(M_{i}\geq M_{obs}\right)}{N+1},$$

The empirical *p*-value was 0.0099. These findings support the presence of a non-random structure–activity signal at the model validation level but do not imply statistical significance for individual compound predictions. Complete permutation-level results are provided in Supplementary Data Sheet S10. Therefore, the permutation analysis supports the presence of a nonrandom predictive signal at the model validation level. This negative control interpretation is consistent with the established QSAR validation principles [39,40,41].

### 2.7. Applicability-Domain Assessment

**Figure 4:**
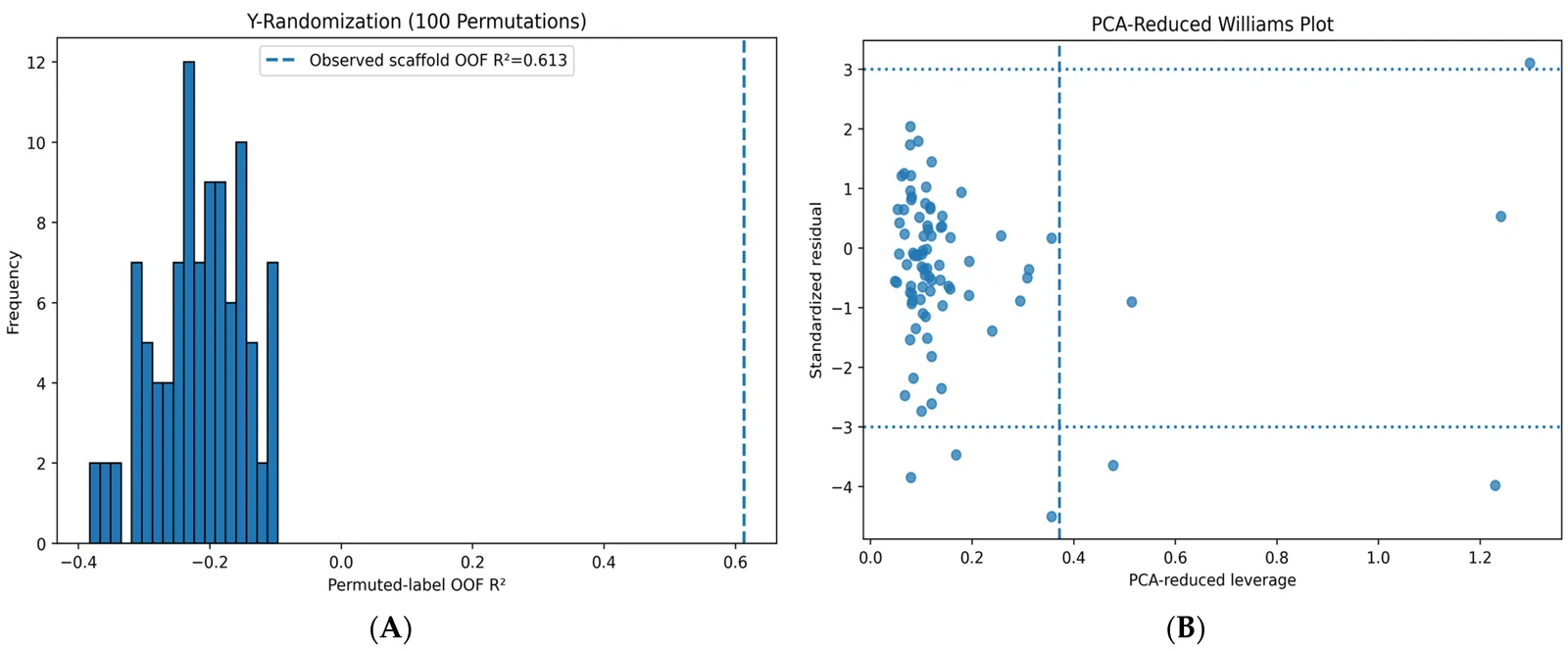
Regression validation and applicability domain. (**A**) Y-randomization distribution relative to the observed scaffold-OOF R^2^; the dashed vertical line indicates the observed scaffold-OOF R^2^. (**B**) PCA-reduced Williams/leverage applicability domain assessment; the dashed vertical line indicates the leverage threshold.

Two complementary applicability domain procedures were applied to the 85 locked regression compounds. The first used standardized descriptor-space distances for the five nearest development compounds. At the predefined 5-NN distance threshold of 23.7783, 76 of 85 compounds (89.4%) were classified as within domain and 9 (10.6%) as outside domain. The second procedure used a PCA-reduced Williams/leverage framework. A total of 41 principal components, explaining approximately 95.05% of the descriptor variance, were retained, with a leverage threshold of h* = 0.3717. Under the combined PCA-Williams criterion, 77 compounds (90.6%) were classified as within the domain and eight (9.4%) as outside. The leverage-only criterion classified 80 compounds within the domain and five outside, whereas the residual-only component classified 79 within and six outside; the combined 77/8 assignment was used as the final PCA-Williams result. The two final applicability domain procedures agreed for 78 of the 85 compounds (91.8%), with seven discordant assignments. The results are shown in Figure 4 and summarized in Table 7. Complete compound-level domain assignments are provided in Supplementary Data Sheet S11. Because the outside-domain groups contained only nine and eight compounds, respectively, subgroup performance estimates—particularly R^2^—were considered unstable and were not used to draw strong comparative conclusions. Applicability domain membership is interpreted as an indicator of structural and descriptor-space support rather than proof of prediction correctness or chemical reliability [45,46,47,48,49,50,51,52].

The PCA-Williams analysis retained 41 components, explaining approximately 95.05% of the descriptor variance. The overall agreement between the two final domain classifications was 78/85 compounds (91.8%).

### 2.8. Scaffold-Disjoint Classification Performance

The primary classification population was independently divided into 292 development compounds representing 98 scaffolds and 73 locked-test compounds representing 45 scaffolds, with no shared Bemis–Murcko scaffolds between them. Compound-level partition assignments are provided in Supplementary Data Sheet S12.

Under five-fold scaffold-grouped cross-validation, the selected Gradient Boosting classifier achieved a mean ROC-AUC of 0.9068 ± 0.1277, PR-AUC of 0.9512 ± 0.0786, balanced accuracy of 0.8147, and MCC of 0.6521. Pooled OOF performance was ROC-AUC = 0.9273, PR-AUC = 0.9763, balanced accuracy = 0.8588, and MCC = 0.6675.

**Figure 5:**
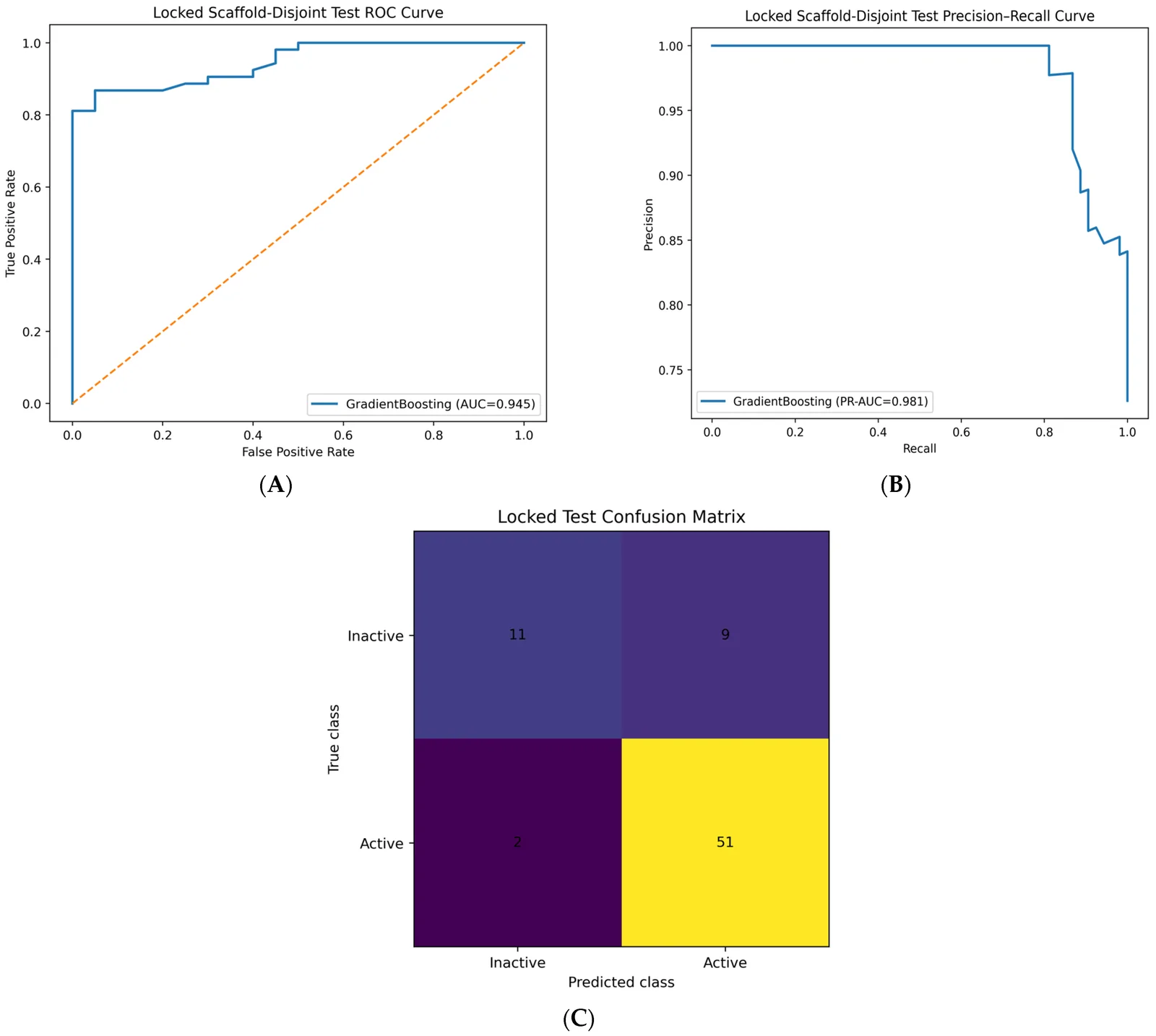
Performance of the frozen scaffold-disjoint FXIIa classification model. (**A**) Receiver operating characteristic curve for the locked test population; the dashed diagonal line represents the random-classifier reference. (**B**) Precision–recall curve. (**C**) Locked-test confusion matrix comprising 11 true negatives, 9 false positives, 2 false negatives, and 51 true positives; color intensity represents the number of compounds in each cell.

On the locked 73-compound scaffold-disjoint test population, the ROC-AUC was 0.9453, and PR-AUC was 0.9807. Accuracy was 0.8493, balanced accuracy 0.7561, and MCC = 0.5972. The MCC is particularly informative for binary classification evaluation when class distributions and class-specific error patterns need to be considered jointly [50,51] (Table 8; Figure 5A,B).

The locked-test confusion matrix comprised 11 true negatives, 9 false positives, 2 false negatives, and 51 true positives (Figure 5C). Therefore, the sensitivity was high (0.9623), whereas the specificity was modest (0.5500). The precision, negative predictive value, and F1 score were 0.8500, 0.8462, and 0.9027, respectively. The complete classification performance outputs are provided in Supplementary Data Sheet S13.

Thus, although ranking-based discrimination remained strong, the balanced accuracy, MCC, and specificity showed that the performance was not uniformly near-perfect across both classes.

Locked test supplementary metrics: accuracy = 0.8493; sensitivity = 0.9623; specificity = 0.5500; precision = 0.8500; NPV = 0.8462; F1 = 0.9027; TN = 11; FP = 9; FN = 2; and TP = 51.

### 2.9. Structural-Similarity Audit of the Classification Test Set

**Figure 6:**
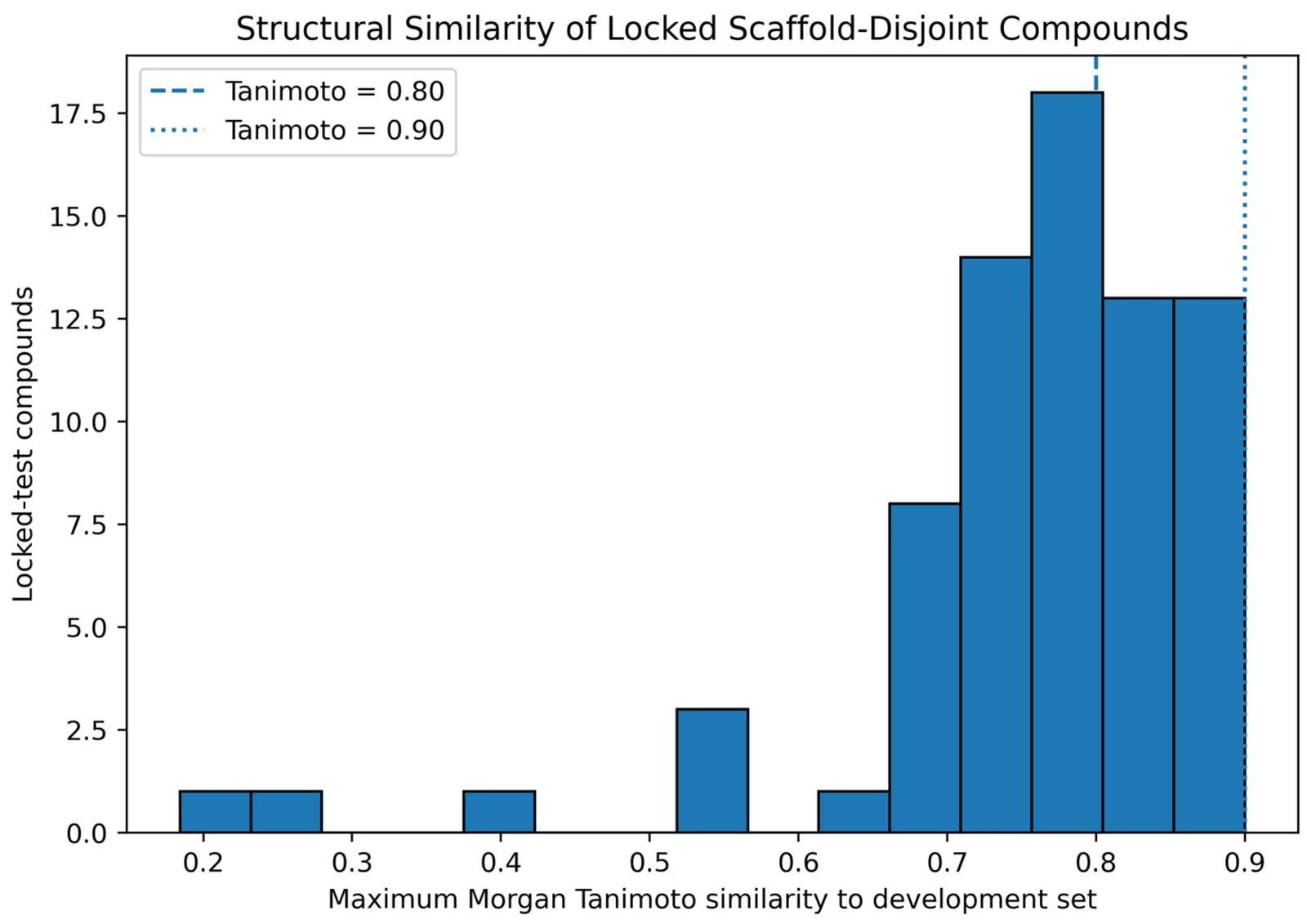
Structural similarity audit of the locked scaffold-disjoint classification population. Maximum Tanimoto similarity to the development population was calculated using RDKit Morgan fingerprints (radius = 2, 2048 bits). Similarity distributions are shown for the complete locked test population and according to prediction correctness.

To determine whether scaffold separation nevertheless left substantial local structural similarity between the development and locked-test compounds, each of the 73 locked classification compounds was compared with the development population using RDKit [52] Morgan fingerprints (radius = 2, 2048 bits), and maximum Tanimoto similarity (Figure 6).

The mean maximum development set similarity was 0.7550, with a median of 0.7816. A total of 30 of the 73 compounds (41.1%) had a maximum similarity ≥ 0.80, whereas only one compound (1.37%) reached 0.90. The maximum similarities ranged from 0.1842 to 0.9000.

The correctly classified compounds had a mean maximum similarity of approximately 0.7705, compared with 0.6676 among the 11 incorrectly classified compounds. This descriptive difference suggests that a lower development set similarity may contribute to more difficult predictions for some compounds, although the relatively small number of errors does not support a formal inferential conclusion.

The similarity audit therefore confirmed that scaffold disjointness did not imply a complete absence of substructural resemblance, while also showing that the locked set was not dominated by near-identical development compounds. Compound-level similarity results are provided in Supplementary Data Sheet S14.

### 2.10. Classification Y-Randomization

**Figure 7:**
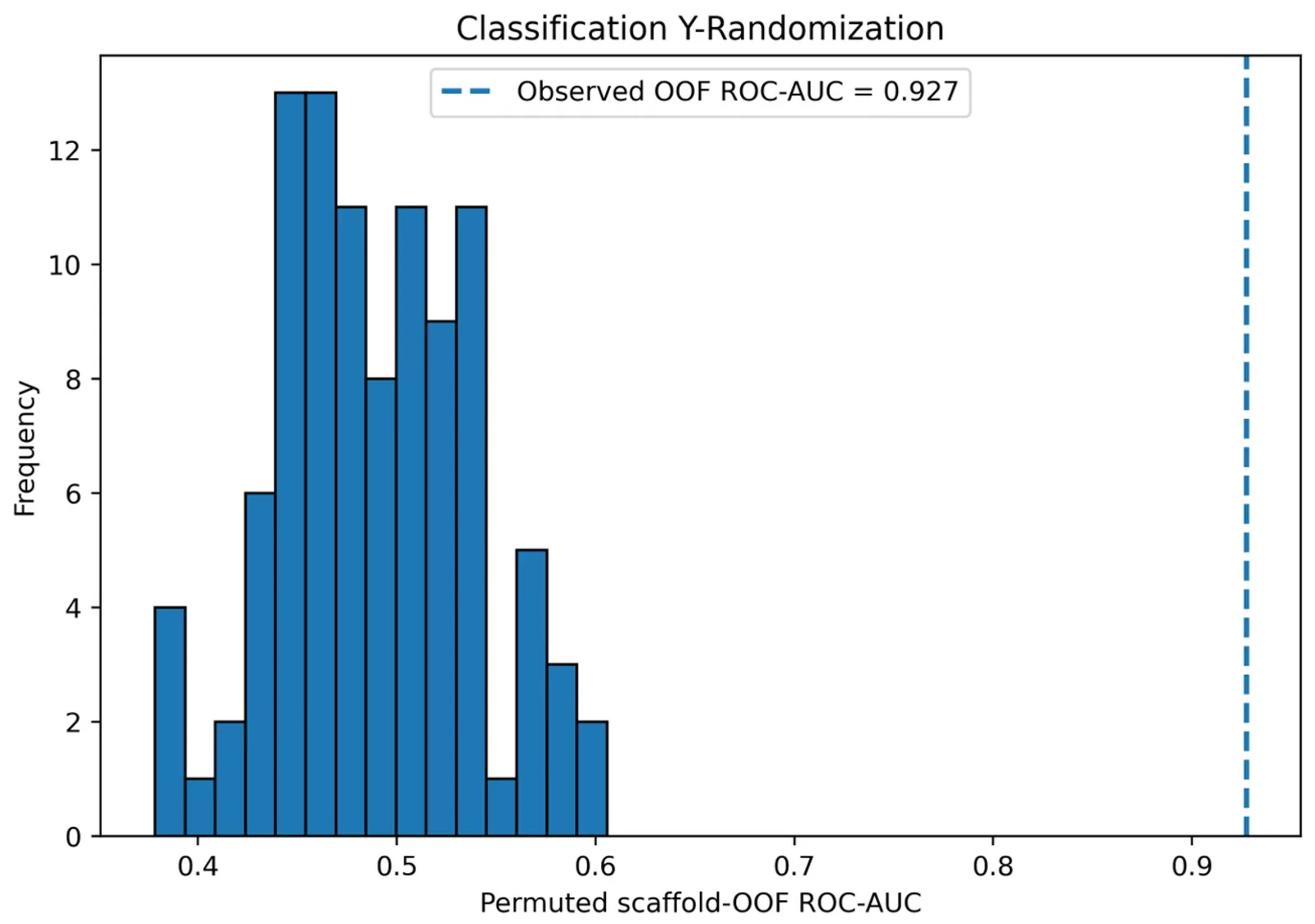
Distribution of scaffold-OOF ROC-AUC values obtained from 100 classification Y-randomizations compared with the observed model performance.

Classification Y-randomization was performed using 100 independent label permutations while preserving a scaffold-aware validation framework (Figure 7).

The observed pooled OOF ROC-AUC was 0.9273, compared with a randomized mean of 0.4889 ± 0.0497. The randomized ROC-AUC values ranged from 0.3783 to 0.6058, and none reached the observed value.

Similarly, the observed pooled OOF MCC was 0.6675, whereas the randomized MCC values had a mean of −0.0071 ± 0.0691 and a maximum of 0.1647. The empirical probability was 0.0099 for both the ROC-AUC and MCC.

These results provide a model-level negative control that supports a nonrandom relationship between molecular representation and classification labels under the scaffold-aware validation framework. The complete classification Y-randomization outputs are provided in Supplementary Data Sheet S15. This interpretation is consistent with the permutation-based controls used in QSAR validation [39,40,41].

### 2.11. SHAP Interpretation, Descriptor Correlation, and Ranking Stability

**Figure 8:**
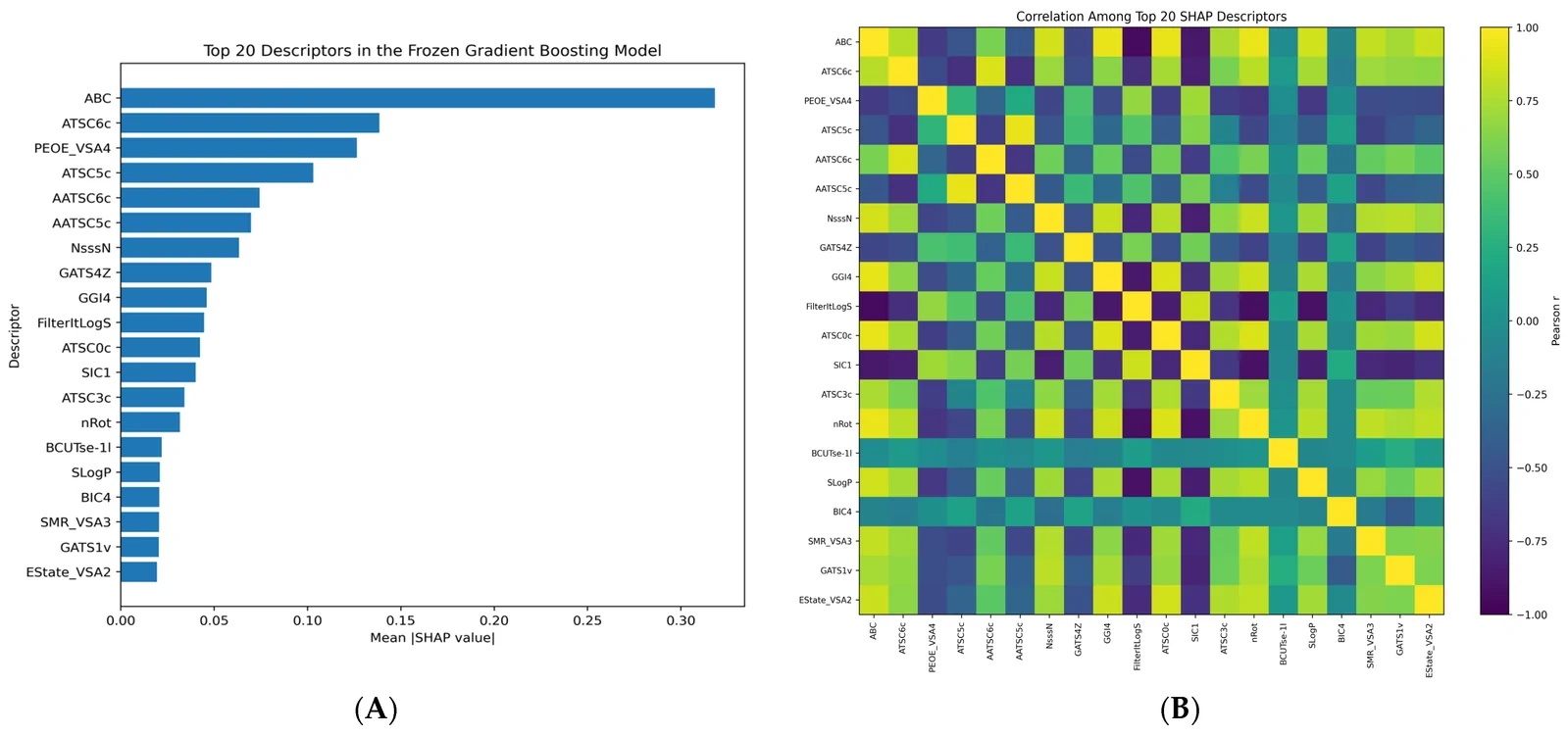

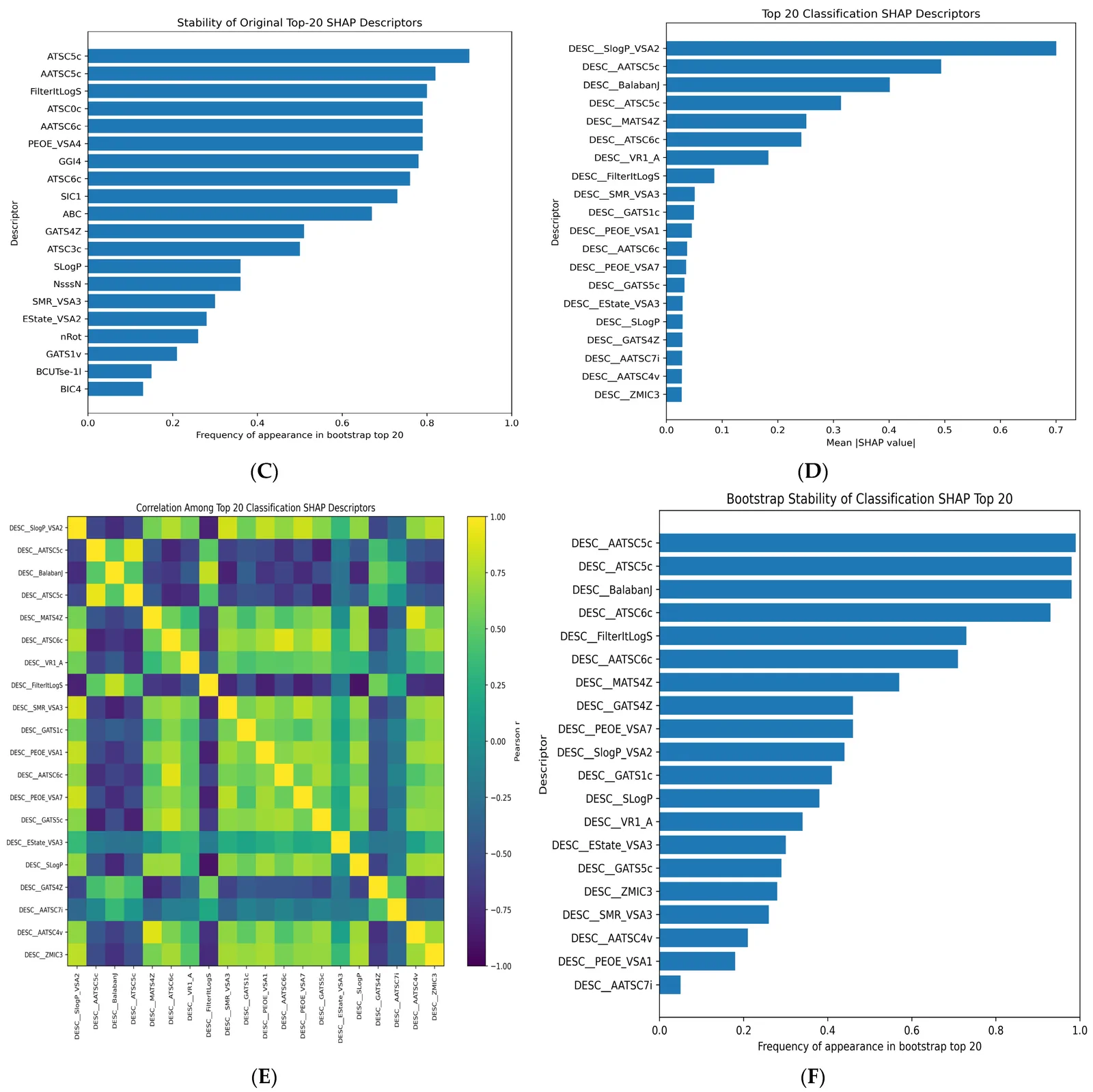
SHAP-based model interpretation and ranking stability for regression and classification models. (**A**) Mean absolute SHAP importance of the top 20 regression descriptors. (**B**) Pairwise Pearson correlations among the top 20 regression SHAP descriptors. (**C**) Bootstrap frequency with which the original top 20 regression descriptors reappeared among the top 20 descriptors across 100 perturbations. (**D**) Mean absolute SHAP importance of the top 20 classification descriptors. (**E**) Pairwise Pearson correlations among the top 20 classification SHAP descriptors. (**F**) Bootstrap frequency with which the original top 20 classification descriptors reappeared among the top 20 descriptors across 100 perturbations.

SHAP analysis [45,46] was used to characterize the descriptors that contributed most strongly to the predictions from frozen regression and classification models (Table 9; Figure 8).

For regression, ABC had the highest mean absolute SHAP value (0.3182; Figure 8A), followed by descriptors, including ATSC6c, PEOE_VSA4, ATSC5c, AATSC6c, and AATSC5c. Among the top 20 regression descriptors, 29 descriptor pairs had |r| ≥ 0.80 and seven had |r| ≥ 0.90 (Figure 8B), demonstrating a substantial correlation among some highly ranked molecular representations.

The overall regression SHAP ranking was highly concordant between the development and lock-test populations (Spearman ρ = 0.9909). However, 100 bootstrap perturbations of the development population produced a mean rank correlation of 0.5148 ± 0.0363, with a median of 0.5165 and an empirical 2.5th–97.5th percentile range of 0.4407–0.5812, indicating good concordance. The mean top-10 and top-20 overlap fractions were approximately 55.3% and 54.45%, respectively (Figure 8C). The complete regression SHAP and ranking stability outputs are provided in Supplementary Data Sheets S16 and S17.

For classification, SlogP_VSA2 had the highest mean absolute SHAP value (0.7003; Figure 8D), followed by AATSC5c, BalabanJ, ATSC5c, and the other descriptors. Among the top 20 classification descriptors, 16 pairs had |r| ≥ 0.80 and three had |r| ≥ 0.90 (Figure 8E).

Development-versus-locked classification SHAP rankings were likewise highly concordant (ρ = 0.9921), whereas bootstrap perturbation produced a mean rank correlation of 0.4252 ± 0.0393, with a median of 0.4302, and an empirical 2.5th–97.5th percentile range of 0.3480–0.5046. The mean top-10 and top-20 overlaps were approximately 54.4% and 49.75%, respectively (Figure 8F). The complete classification SHAP and ranking stability outputs are provided in Supplementary Data Sheets S16 and S17.

Together, these findings indicate that broad attribution patterns were highly consistent between the frozen development and locked-test populations, whereas the exact ordering of individual descriptors was only moderately stable under the bootstrap perturbation.

## 3. Discussion

### 3.1. Overall Predictive Performance and Chemical-Series Generalization

The present study developed an assay-context-defined, IC50-specific, machine-learning framework for predicting human FXIIa activity. Restricting the primary dataset to assays explicitly referring to FXIIa, Factor XIIa, or activated Factor XII reduced the ambiguity between the formal ChEMBL target annotation and the experimentally described target state. This distinction is important because retrospective bioactivity databases integrate measurements obtained under heterogeneous assay conditions, and IC50 values remain dependent on the experimental design even after endpoint-specific curation [29,30,31,32,33,34].

The selected Gradient Boosting regressor achieved R^2^ = 0.7560, RMSE = 0.6924, and MAE = 0.4965 on the locked scaffold-disjoint test population (Table 5; Figure 3A; Supplementary Data Sheet S8). These results indicate useful predictive performance across non-overlapping Bemis–Murcko scaffolds. However, the locked population originated from the same curated ChEMBL-derived dataset as the development population and should, therefore, be interpreted as an internal scaffold-disjoint holdout rather than an independent external validation set.

Fifty repeated scaffold-disjoint evaluations provided an important complementary perspective. The mean R^2^ was 0.6892 ± 0.1515, compared with R^2^ = 0.7560 for the frozen locked partition, while the empirical 2.5th–97.5th percentile range extended from 0.3571 to 0.8501 (Table 5; Figure 3B; Supplementary Data Sheet S9). The variability across partitions indicates that the predictive performance depends partly on the chemical series assigned to the development and evaluation populations. Therefore, a single scaffold-disjoint split does not fully characterize generalization.

This observation is consistent with the broader concerns surrounding chemically structured validation. Although scaffold splitting is generally more demanding than random compound-level partitioning, it does not guarantee a uniform evaluation difficulty or complete structural independence between the development and test populations [53]. Accordingly, the locked test result and repeated scaffold-disjoint distribution should be interpreted together rather than treating either statistic as a complete measure of prospective performance.

The relatively similar development-set performances of Gradient Boosting, XGBoost, and Extra Trees provide additional context (Table 4; Supplementary Data Sheets S6 and S7). Therefore, the principal finding is that a single ensemble architecture is not universally superior for FXIIa activity prediction, but that nonlinear descriptor-based models capture useful structure–activity information under chemically structured and leakage-controlled evaluations.

### 3.2. Negative Controls and Applicability-Domain Support

Y-randomization provided a complementary negative control for the regression analysis. The observed scaffold-grouped OOF R^2^ of 0.6130 was clearly separated from the 100 randomized models, which produced a mean R^2^ of −0.2157 ± 0.0688 and a maximum value of −0.0964. None of the randomized models equaled or exceeded the observed statistics, yielding an empirical *p* = 0.0099 (Table 6; Figure 4A; Supplementary Data Sheet S10).

These results support the presence of a nonrandom predictive signal at the model validation level. However, they should not be interpreted as establishing statistical certainty or experimental correctness for individual compound predictions. Y-randomization evaluates whether a comparable validation performance can arise after the disruption of the structure–activity relationship; however, it does not provide compound-specific confidence estimates [35,36,37,38].

Applicability domain analyses address different aspects of prediction support. The standardized-space 5-NN approach classified 76 of the 85 locked compounds (89.4%) as within-domain, whereas the combined PCA-Williams procedure classified 77 of the 85 compounds (90.6%) as within-domain (Table 7; Figure 4B; Supplementary Data Sheet S11). The agreement between the two assignments was 78/85 (91.8%).

The high agreement provides convergent evidence regarding representation support; however, applicability domain membership should not be interpreted as a direct measure of chemical or experimental reliability. Established applicability domain frameworks emphasize that domain assignments depend on molecular representation, distance or leverage definitions, and the statistical procedure used to characterize the modeled space [42,43,44]. Therefore, the two complementary approaches used here provide a structural and descriptor-space context for the locked predictions rather than guarantees of predictive accuracy.

Only nine compounds were classified as outside the domain by the 5-NN criterion and eight by the combined PCA-Williams criterion. These subsets are too small to support stable subgroup R^2^ estimates. Consequently, negative or highly variable R^2^ values calculated from such small subsets should not be interpreted as evidence of model calibration failure. The RMSE and MAE remain more interpretable descriptive measures when examining prediction errors in very small subsets.

Y-randomization and applicability domain assessment thus address complementary but distinct questions: the former evaluates whether the overall predictive relationship exceeds that obtained after response randomization, whereas the latter evaluates whether query compounds are represented within the molecular space supporting model development.

### 3.3. Classification Performance, Threshold Dependence, and Structural Similarity

The primary classification model showed strong ranking performance on the locked scaffold-disjoint population, with ROC-AUC = 0.9453 and PR-AUC = 0.9807 (Table 8; Figure 5; Supplementary Data Sheets S12 and S13). The balanced accuracy (0.7561) and MCC (0.5972) provided a more conservative assessment of the overall binary performance [51]. The confusion matrix revealed high sensitivity (0.9623) but lower specificity (0.5500), indicating that inactive compounds were more difficult to identify than active compounds.

The distinction between ranking and threshold-dependent metrics is important in the present dataset. High ROC-AUC and PR-AUC values indicate effective ranking of compounds across predicted scores, whereas lower balanced accuracy and MCC reflect errors at the frozen classification decision boundary. Therefore, the classification performance should not be summarized solely using the comparatively high ROC-AUC or PR-AUC values.

Because the primary pIC50 < 6.0 versus ≥ 7.0 boundaries were operational modeling definitions rather than universal biological thresholds, alternative activity definitions were explicitly examined (Table 2; Supplementary Data Sheet S4). The ROC-AUC remained comparatively stable across the evaluated scenarios, whereas the balanced accuracy and MCC showed greater variation. For example, the active ≥ 7.0/inactive < 6.5 scenario yielded a balanced accuracy of 0.8061 and MCC of 0.6758, whereas active ≥ 6.5/inactive < 6.0 produced values of 0.7071 and 0.5185, respectively. These differences illustrate that the apparent binary performance depends partly on how intermediate-potency compounds are handled.

Importantly, these analyses did not retrospectively redefine scaffold splits. The primary frozen scaffold allocation was preserved, and compounds newly admitted from the original gray zone were evaluated only when their scaffolds already possessed a development/test assignment. This constraint prevented alternative thresholds from generating new partitions that could be inadvertently influenced by the locked-test performance.

Classification Y-randomization provided an additional negative control. The observed pooled OOF ROC-AUC of 0.9273 and MCC of 0.6675 exceeded all corresponding values from the 100 randomized datasets, yielding empirical *p* = 0.0099 for both metrics (Figure 7; Supplementary Data Sheet S15). As in regression, these findings support a non-random predictive structure at the model-validation level rather than statistical certainty for individual classifications.

The structural similarity audit provided further context for the scaffold-disjoint classification results. The mean maximum development-set Tanimoto similarity of the locked compounds was 0.7550, and 30 of the 73 compounds (41.1%) had a maximum similarity of ≥0.80. In contrast, only one compound reached a correlation coefficient of ≥0.90 (Figure 6; Supplementary Data Sheet S14). Correctly classified compounds had a descriptively higher mean maximum similarity than incorrectly classified compounds (0.7705 versus 0.6676), although no inferential significance tests were applied.

These findings emphasize that scaffold disjoint does not imply structural irrelevance. Molecules assigned to different Bemis–Murcko frameworks can retain substantial local or fingerprint-level similarities. This distinction is consistent with reports that scaffold-based splits may still overestimate performance in some virtual screening settings [53]. The present classification results, therefore, support the prediction across non-overlapping scaffolds within the represented FXIIa chemical domain, rather than unrestricted generalization to arbitrary or substantially different chemical matter.

### 3.4. SHAP Interpretation and Descriptor-Ranking Stability

SHAP analysis identified distinct model-influential descriptor profiles for both regression and classification [45,46]. ABC had the highest mean absolute SHAP value for regression (0.3182), whereas SlogP_VSA2 ranked highest for classification (0.7003) (Table 9; Figure 8A,D; Supplementary Data Sheets S16–S17).

These rankings should not be interpreted as direct biological or mechanistic determinants of FXIIa activity in the plasma. Molecular descriptors are mathematical representations of structural or physicochemical properties, and correlated descriptors may encode overlapping data. Among the top 20 SHAP-ranked variables, 29 regression descriptor pairs showed |r| ≥ 0.80, including seven at ≥0.90, whereas 16 classification pairs exceeded |r| ≥ 0.80 and three exceeded ≥ 0.90 (Figure 8B,E).

This correlation structure is particularly relevant for interpretation because attribution can be distributed among descriptors carrying related information. Therefore, a high SHAP ranking establishes that a descriptor influences the predictions within the fitted model but does not establish that the represented molecular property independently controls FXIIa activity.

Development-versus-locked SHAP rankings were highly concordant, with Spearman ρ = 0.9909 for regression and ρ = 0.9921 for classification. However, bootstrap perturbation of the development population produced substantially more moderate ranking stability: the mean Spearman ρ was 0.5148 ± 0.0363 for regression and 0.4252 ± 0.0393 for classification. The mean top-20 overlaps were 54.45% and 49.75%, respectively (Table 9; Figure 8C,F; Supplementary Data Sheets S16 and S17).

Taken together, these results indicate that the broad attribution patterns were highly concordant between the frozen development and locked populations, whereas the exact ordering of individual descriptors showed only moderate stability under sampling perturbations. Therefore, SHAP interpretation should focus on broader model-influential descriptor patterns rather than assigning mechanistic importance to small differences in individual feature ranks [45,46].

### 3.5. Relationship to Previous QSAR Studies and Methodological Contribution

The present study should be considered in the context of previous computational modeling studies of FXIIa and related coagulation targets. Chen and Visco developed PCA-GA-SVM models for virtual high-throughput screening of FXIIa inhibitors [47], demonstrating that ligand-based computational approaches can capture structure–activity relationships relevant to this target

Our previous studies have similarly applied machine learning QSAR approaches to Factor X and FXIa [48,49]. These studies collectively demonstrate the applicability of descriptor-based machine learning across multiple coagulation-related targets. However, direct numerical comparisons among their reported performance statistics are inappropriate because the underlying compound populations, assay endpoints, target contexts, molecular representations, preprocessing procedures, and validation strategies differ.

Accordingly, the contribution of the present study does not rest on claiming the first QSAR model for FXIIa or demonstrating numerical superiority over previous models. The principal methodological contribution is the integration of assay-context-defined target selection, IC50-specific curation, model-independent scaffold partitioning, leakage-safe preprocessing, locked and repeated scaffold-disjoint evaluation, Y-randomization, complementary applicability-domain analyses, classification-threshold sensitivity, structural-similarity auditing, and SHAP ranking-stability assessment within a single workflow.

This combination of analyses was designed to distinguish predictive performance from the conditions under which the performance was obtained. In particular, repeated scaffold analysis characterizes dependence on chemical-series allocation; Y-randomization evaluates non-random predictive signals; applicability domain procedures describe representation support; structural similarity analysis quantifies residual similarity despite scaffold separation; and SHAP bootstrap analysis evaluates the stability of interpretation. Therefore, these components address complementary sources of uncertainty rather than serving as interchangeable indicators of model validity.

### 3.6. Biological and Translational Context

FXIIa occupies an intersection between contact activation, coagulation, kallikrein–kinin signaling, and inflammatory processes [2,3,6,7,8,9]. Genetic and pharmacological interference with FXII/FXIIa reduces pathological thrombus formation in several preclinical systems [4,10,11,12,13,14,20,21,22]. These observations provide a biological rationale for the continued investigation of FXIIa as a pharmacological target, while the broader involvement of the contact system in inflammatory and bradykinin-mediated pathways argues against reducing its biological significance to thrombosis alone [6,7,8,9,15,16,17,18,19].

The clinical development of garadacimab further demonstrates that FXIIa can be pharmacologically targeted in human beings. In the phase III VANGUARD trial, garadacimab reduced the number of attacks of hereditary angioedema [18]. However, hereditary angioedema is predominantly a bradykinin-mediated disease, and these clinical findings should therefore be interpreted as evidence of FXIIa target tractability rather than direct clinical validation of an antithrombotic strategy for FXIIa.

The discovery of small-molecule FXIIa has also progressed substantially, with studies reporting multiple inhibitor chemotypes and increasingly potent, selective, and pharmacologically developed compounds [23,24,25,26,27,28]. The present models may complement these experimental approaches by supporting the computational prioritization of compounds before biochemical evaluation.

Importantly, the predicted pIC50 values, classification probabilities, applicability domain assignments, and SHAP patterns should not be interpreted as evidence of therapeutic efficacy. Their intended role is to prioritize compounds for subsequent experimental investigations within the chemical and assay domains represented by the training data.

### 3.7. Limitations and Future Directions

The present study has several limitations. First, although restricting exact IC50 measurements from assays explicitly referring to FXIIa improved endpoint and target-context consistency, the dataset remained retrospective and experimentally heterogeneous. IC50 measurements may vary depending on the substrate concentration, incubation time, enzyme concentration, assay format, and inhibition mechanism [29,30,31]. The available ChEMBL records do not provide sufficiently uniform information on these variables to establish complete assay equivalence.

Second, the primary regression dataset contained 424 compounds representing 166 scaffolds, and repeated scaffold-disjoint assessments demonstrated meaningful sensitivity to chemical-series allocation. Therefore, the frozen locked population represents one chemically separated realization within the available dataset rather than a complete estimate of performance across the entire FXIIa chemical space.

Third, both regression and classification-locked populations originated from the same curated ChEMBL-derived dataset as their respective development populations. Therefore, they should be regarded as internal scaffold-disjoint holdouts and not independent external experimental validations. Prospective measurements of newly synthesized compounds would provide a substantially stronger test of model transferability.

Fourth, the classification performance was influenced by the operational activity thresholds, and the primary locked model showed substantially lower specificity than sensitivity (Table 2 and Table 8). Threshold sensitivity analysis reduces dependence on an arbitrary activity definition but does not establish a universal biological boundary between active and inactive FXIIa compounds.

Fifth, scaffold separation does not eliminate residual molecular similarities. Approximately 41% of the locked classification compounds had a maximum development set Tanimoto similarity of ≥0.80 (Figure 6; Supplementary Data Sheet S14). Consequently, scaffold-disjoint performance should not be interpreted as evidence of unrestricted extrapolation to a structurally remote chemical space [53].

Sixth, Y-randomization and applicability domain analyses have distinct and limited interpretations. Permutation testing supports a nonrandom predictive signal at the model validation level, whereas applicability domain membership describes representation support [42,43,44]. Neither establishes experimental accuracy for an individual compound.

Seventh, the SHAP interpretation remains model-dependent [45,46]. The correlation among highly ranked descriptors and only moderate bootstrap stability of exact feature ordering indicate that individual descriptors should not be assigned causal biological significance (Table 9; Figure 8; Supplementary Data Sheets S16 and S17).

Finally, the exact installed Mordred package version was not retained during the computational workflow and, therefore, should not be reconstructed retrospectively. The descriptor implementation itself is documented by the cited Mordred methodology [50], while all other retained software versions, random seeds, frozen partitions, and principal modeling parameters are reported in the Supplementary Data Sheet S18.

Therefore, the most important next step is prospective evaluation using independently generated FXIIa activity measurements under standardized experimental conditions. Candidate selection can integrate predicted potency, classification probability, applicability domain status, and structural novelty. Experimental testing of such candidates would provide a substantially stronger assessment of predictive transferability and practical utility than the additional retrospective partitioning of the current ChEMBL-derived dataset.

## 4. Materials and Methods

### 4.1. Data Collection and Endpoint Curation

Bioactivity records associated with the human single-protein target CHEMBL2821, formally annotated in ChEMBL as *Coagulation factor XII*, were retrieved from ChEMBL release 37 on 28 August 2026 [32]. Because the present study specifically addressed the activated protease form FXIIa, target assignment was further refined at the assay level. Primary modeling was restricted to assays whose available descriptions explicitly referred to “FXIIa,” “Factor XIIa,” or “activated Factor XII.” This assay-text criterion was used as a conservative operational definition of the FXIIa modeling domain and was not intended to imply that assays lacking these explicit terms necessarily measured a biologically distinct target state.

Only the IC50 records were considered for primary modeling. Eligible activity records were required to have an exact relation (=), nanomolar units, positive activity values, no ChEMBL activity validity warning, and a valid standardized molecular structure. Ki and other potency endpoints were not pooled with IC50 because these measurements are not experimentally interchangeable, and their combination may introduce heterogeneity arising from non-equivalent endpoint definitions [29,30,31].

The IC50 values were converted to pIC50 as follows:

(2) $${pIC}_{50}=9-{log}_{10}\left({IC}_{50}\left[nM\right]\right)$$

Replicate measurements associated with the same standardized compounds were consolidated using the median pIC50. Replicate variability was audited independently rather than being used as a universal exclusion criterion. Four compound groups in the broader curated population showed replicate ranges > 1 pIC50 unit, of which two occurred within the explicit FXIIa primary population. These compounds were retained in the primary analysis; their exclusion generated a 422-compound replicate-strict sensitivity population.

After assay context restriction and replicate consolidation, the primary regression dataset comprised 424 compounds representing 166 Bemis–Murcko scaffolds. No additional exclusion based on physicochemical properties, peptide-like characteristics, or presumed lead-likeness was applied. Detailed compound-level curation and replicate auditing are provided in Supplementary Data Sheets S1 and S2.

### 4.2. Endpoint Definition and Molecular Descriptors

All 424 compounds were retained for the continuous regression modeling. For the primary binary classification task, compounds with pIC50 ≥ 7.0 were defined as active, and those with pIC50 < 6.0 were defined as inactive. Compounds with intermediate activity were assigned to a gray zone and excluded from the primary binary analysis.

(3) $$y_{i}=\left\{\begin{array}{cc} 0, & {pIC}_{50}<6.0\\ 1, & {pIC}_{50}\geq 7.0 \end{array}\right.$$

This definition produced 267 active and 98 inactive compounds (*n* = 365), and 59 compounds were assigned to the gray zone. Because these thresholds represent operational modeling definitions rather than universal biological boundaries, alternative definitions were evaluated separately in sensitivity analyses (Supplementary Data Sheets S3 and S4).

Two-dimensional molecular descriptors were calculated using Mordred [50], with three-dimensional descriptors being disabled. The initial descriptor matrix contained 1613 variables. Descriptor availability and variability were inspected descriptively at the full dataset stage; however, no global feature-selection result was transferred into predictive modeling. All data-dependent descriptor filtering used for model development was learned exclusively from the corresponding development data points.

### 4.3. Scaffold-Disjoint Partitioning and Leakage-Safe Preprocessing

The Bemis–Murcko frameworks [41] were used as scaffold identifiers. For regression, 2000 candidate scaffold-disjoint partitions were generated and ranked using a predefined model-independent balance criterion based only on the test set fraction and pIC50-distribution characteristics. The recorded diagnostics included development/test sample sizes, scaffold counts, activity means, medians and standard deviations, distribution differences, and the Kolmogorov–Smirnov statistic. No molecular descriptor or predictive model performance was used to select the locked split.

The selected regression partition was candidate 1952 (seed = 20,262,779), comprising 339 development compounds from 114 scaffolds and 85 locked-test compounds from 52 scaffolds with zero shared scaffolds. The development and locked-test mean pIC50 values were 6.9666 and 6.9423, respectively, and the corresponding distributions yielded a two-sample Kolmogorov–Smirnov *p*-value of 0.9922. The split details are provided in Table 3 and Supplementary Data Sheet S5.

All data-dependent preprocessing was restricted to the development data. The frozen preprocessing sequence consisted of the following: removal of descriptors with >20% missingness in the development population; removal of descriptors with variance ≤ 1 × 10^−12^; removal of redundant descriptors using pairwise Pearson correlation at |r| > 0.95; median imputation; and standardization using development-derived parameters.

For the frozen regression partition, this sequence reduced the representation from 1613 descriptors to 583 descriptors. Importantly, during the five-fold cross-validation, the complete preprocessing procedure was independently refitted within each training fold rather than reusing the preprocessing learned from the full development population. The resulting fold-specific descriptor counts were 591, 589, 580, 570, and 572, respectively.

For classification, 3000 model-independent candidate scaffold partitions were assessed. The frozen primary classification split was candidate 284 (seed = 20,261,115), comprising 292 development compounds from 98 scaffolds and 73 locked-test compounds from 45 scaffolds, with zero shared scaffolds. The classification partition information is provided in Supplementary Data Sheet S12.

### 4.4. Regression Model Development and Performance Assessment

Nine predefined regression algorithms were initially benchmarked using five-fold GroupKFold cross-validation with the Bemis–Murcko scaffold groups. Among these algorithms, Extra Trees [54], XGBoost, and Gradient Boosting [55] produced the strongest development-set results and were advanced to the predefined configuration evaluation.

For each of these three algorithms, 12 configurations were specified prior to the final model selection. Evaluation across the five scaffold-grouped folds comprised 3 × 12 × 5 = 180 fold-level model fits. Configuration selection was based exclusively on the development set cross-validation results; the locked test population was not used for model or hyperparameter selection.

The selected Gradient Boosting regressor used 300 estimators, learning rate = 0.03, maximum depth = 3, minimum samples split = 4, minimum samples leaf = 3, subsample = 0.9, max_features = None, and Huber loss, with random_state = 20,260,828. Benchmark results and frozen model parameters are reported in Supplementary Data Sheets S6–S7 and S18.

The regression performance was quantified using the coefficient of determination:

(4) $$R^{2}=1-\frac{\sum_{i}(\left.y_{i}-{\hat{y}}_{i}{\left.)\right.}^{2}\right.}{\sum_{i}(\left.y_{i}-\bar{y}{\left.)\right.}^{2}\right.}$$

(5) $$RMSE=\sqrt{\frac{1}{n}\sum_{i}(\left.y_{i}-{\hat{y}}_{i}{\left.)\right.}^{2}\right.},MAE=\frac{1}{n}\sum_{i}|y_{i}-{\hat{y}}_{i}|$$

After model and configuration selection, the frozen preprocessing/model pipeline was fitted to the complete 339-compound development population and evaluated once on the 85-compound locked scaffold-disjoint test population.

The uncertainty around the locked-test performance was characterized using 5000 paired bootstrap resamples of the observed/predicted pairs without model refitting. In addition, the sensitivity to chemical-series allocation was evaluated using 50 repeated scaffold-disjoint partitions. The results of these analyses are presented in Table 5 and Supplementary Data Sheets S8 and S9.

### 4.5. Regression Negative Controls and Applicability Domain

Regression Y-randomization was performed using 100 independent response permutations within a scaffold-aware validation framework. For each permutation, the relationship between the molecular descriptors and experimental activity was disrupted while retaining the validation procedure. The observed pooled OOF R^2^ was compared with a randomized distribution.

The empirical probability is calculated as follows:

(6) $$p_{emp}=\frac{1+\sum_{i=1}^{N}I\left(M_{i}\geq M_{obs}\right)}{N+1}$$

where $M_{obs}$ denotes the observed validation metric, $M_{i}$ the corresponding metric from the ith randomized analysis, and N=100. Y-randomization was interpreted as a model-level negative control rather than a compound-specific confidence measure (Supplementary Data Sheet S10).

The applicability domain support was evaluated using two complementary approaches [42,43,44]. First, a standardized descriptor-space 5-nearest-neighbor (5-NN) analysis was performed using a frozen distance threshold of 23.7783 Å.

Second, a PCA-Williams analysis was conducted using 41 principal components, which explained 95.05% of the development set variance. Warning leverage is defined as

(7) $$h^{*}=\frac{3\left(k+1\right)}{n}$$

where k is the number of retained principal components and n is the number of development compounds. This resulted in *h* = 0.3717*.

Leverage and standardized residual information were jointly considered for the principal PCA-Williams domain assignment. Leverage-only and residual-only classifications were retained as diagnostic support. Applicability domain membership was interpreted as representation support within the modeled descriptor space, not as proof of chemical or experimental reliability. The full results are provided in Table 7 and Supplementary Data Sheet S11.

### 4.6. Classification Modeling and Threshold Sensitivity

Seven predefined classification algorithms were evaluated using five-fold scaffold-grouped cross-validation: Logistic Regression, RBF-SVC, Random Forest [56,57], Extra Trees [54], Gradient Boosting [55], HistGradientBoosting, and XGBoost.

The primary model selection criterion was the mean MCC, followed sequentially by the mean balanced accuracy and mean ROC-AUC [51]. This hierarchy was specified independently of the locked test performance.

The selected Gradient Boosting classifier used 200 estimators, learning rate = 0.03, maximum depth = 3, minimum samples leaf = 3, minimum samples split = 2, subsample = 0.9, max_features = None, and log-loss objective, with random_state = 20,260,831.

Performance was evaluated using ROC-AUC, PR-AUC, accuracy, balanced accuracy, MCC, sensitivity, specificity, precision, negative predictive value, F1 score, and confusion-matrix counts. The development and locked-test results are provided in Table 8 and Supplementary Data Sheets S12 and S13.

To assess the dependence on the primary activity definition, four alternative threshold scenarios were examined: (i) active ≥ 6.5/inactive < 6.0; (ii) active ≥ 7.0/inactive < 6.5; (iii) a single cutoff at 6.5; and (iv) an expanded gray zone of 5.5–7.0.

These sensitivity analyses preserved the frozen scaffold allocation established from the primary 365-compound classification population. Compounds newly admitted from the original gray zone were included only when their scaffolds already had a frozen development/test assignment. Compounds belonging to scaffolds absent from the primary classification population were omitted rather than re-partitioned.

Accordingly, the threshold-eligible/analyzed sample sizes were 365/365, 405/389, 384/376, 424/400, and 345/345, respectively. No alternative threshold definition was selected according to the locked-test performance (Table 2; Supplementary Data Sheet S4).

### 4.7. Structural-Similarity Audit and Classification Y-Randomization

The residual molecular similarity between the locked classification population and development compounds was evaluated using RDKit [52] Morgan fingerprints with a radius of 2 and 2048 bits. Morgan fingerprints (radius = 2; 2048 bits) and Tanimoto similarity

(8) $$T\left(A,B\right)=\frac{|A\cap B|}{|A|+|B|-|A\cap B|}$$

For each locked compound, the maximum Tanimoto similarity to any of the development compounds was retained. Similarity levels ≥ 0.80 and ≥0.90 were summarized descriptively; these values were not treated as experimentally validated thresholds for predicting reliability. The structural similarity results are shown in Figure 6 and Supplementary Data Sheet S14.

Classification Y-randomization was performed independently using 100 label permutations. Pooled OOF ROC-AUC and MCC were used as reference statistics, and empirical probabilities were calculated according to Equation (6). The results are presented in Figure 7 and Supplementary Data Sheet S15.

### 4.8. SHAP Interpretation and Stability Analysis

Model interpretation was performed using SHapley Additive exPlanations (SHAP) [45] and TreeExplainer [46]. The global descriptor importance was defined using the development-set to mean absolute SHAP magnitude as follows:

(9) $$I_{j}=\frac{1}{n}\sum_{i=1}^{n}|\phi_{ij}|$$

Pairwise Pearson correlations among the top 20 SHAP-ranked descriptors were calculated using the development population, with |r| ≥ 0.80 and ≥0.90 used as descriptive indicators of strong descriptor correlation.

Locked-test SHAP rankings were used only for descriptive comparison with development set rankings and did not contribute to the model or feature selection.

Ranking stability was evaluated using 100 bootstrap perturbations of the development compounds sampled with their replacement. For each perturbation, the SHAP rankings were compared with the original development-set ranking. Stability measures included the Spearman rank correlation, top-10 overlap, top-20 overlap, and descriptor-specific top-10 and top-20 frequencies.

The SHAP results were interpreted as measures of model-influential descriptor attribution, not as evidence of causal molecular mechanisms. The regression and classification SHAP analyses are provided in Table 9, Figure 8, and Supplementary Data Sheets S16 and S17.

### 4.9. Software and Reproducibility

Analyses were performed using Python 3.13.15, NumPy 2.1.3, pandas 2.2.3, SciPy 1.16.3, scikit-learn 1.6.1 [58], RDKit 2026.03.5 [52], SHAP 0.47.2 [45,46], XGBoost 3.4.1, and joblib 1.5.3. Mordred was used for the descriptor generation [50].

The exact installed Mordred package version was not retained during the computational workflow and, therefore, was not inferred retrospectively. Frozen partition identifiers, random seeds, model configurations, preprocessing parameters, validation settings, and retained software version information are provided in Supplementary Data Sheet S18.

### 4.10. Data Availability

The data supporting the findings of this study are provided in the Supporting Information section of this article. The accompanying FXIIA Supplementary Data workbook contains the curated compound-level data, replicate audit, frozen scaffold assignments, model outputs, validation analyses, applicability domain results, structural-similarity audit, SHAP analyses, and methodological parameters across Supplementary Data Sheets S1–S18.

## 5. Conclusions

In this study, we developed scaffold-aware and interpretable machine-learning QSAR models for human FXIIa activity using an endpoint-specific and assay-context-defined ChEMBL dataset. The restriction to exact IC50 measurements from assays explicitly referring to FXIIa yielded a primary regression population of 424 compounds representing 166 Bemis–Murcko scaffolds, providing a clearly defined chemical and experimental domain for model development.

The selected Gradient Boosting regression model achieved R^2^ = 0.7560, RMSE = 0.6924, and MAE = 0.4965 on the locked scaffold-disjoint test set. Across 50 repeated scaffold-disjoint partitions, the mean R^2^ was 0.6892 ± 0.1515, demonstrating a useful predictive signal while also revealing a meaningful dependence on chemical-series allocation. The corresponding scaffold-disjoint classification model achieved ROC-AUC = 0.9453, PR-AUC = 0.9807, balanced accuracy = 0.7561, and MCC = 0.5972 on the locked test set, respectively. Threshold sensitivity analysis further showed that ranking performance remained comparatively stable across alternative activity definitions, whereas balanced accuracy and MCC were more dependent on class boundaries.

Both regression and classification Y-randomization yielded empirical *p* = 0.0099, supporting non-random predictive signals at the model validation level. Complementary applicability domain analyses, structural similarity auditing, and SHAP-based interpretation provide additional context for prediction support and model behavior. Although development-versus-locked SHAP attribution patterns were highly concordant, bootstrap analyses showed only moderate stability of the exact descriptor-ranking order, supporting the interpretation of these features as model-influential descriptor patterns rather than causal molecular determinants.

Collectively, these findings support the use of the developed models for the computational prioritization of compounds within the FXIIa chemical and assay domains. However, the locked scaffold-disjoint test sets remain internal to the available ChEMBL-derived dataset and should not be considered an independent external experimental validation. Therefore, prospective evaluation using independently synthesized and experimentally characterized compounds is required to establish predictive transferability beyond the present chemical domain and to determine the practical value of the models for future FXIIa-focused compound discovery.

## Figures and Tables

**Table 1:** Characteristics of the final assay-context-defined FXIIa dataset.

Characteristic	Value
ChEMBL target identifier	CHEMBL2821
ChEMBL target name	Coagulation factor XII
Target type	SINGLE PROTEIN
Organism	*Homo sapiens*
ChEMBL release	37
Retrieval date	28 August 2026
Primary activity endpoint	IC50
Modeled activity	pIC50
Regression compounds	424
Unique Bemis–Murcko scaffolds	166
Mean pIC50	6.9617
Median pIC50	7.3111
SD of pIC50	1.3713
Replicate-strict sensitivity subset	422
Active compounds, pIC50 ≥ 7.0	267
Inactive compounds, pIC50 < 6.0	98
Gray-zone compounds, 6.0 ≤ pIC50 < 7.0	59
Primary binary-classification compounds	365

**Table 2:** Sensitivity of locked scaffold-disjoint classification performance to alternative activity definitions.

Classification Definition	Threshold-Eligible *n*	Analyzed *n*	Development *n*	Locked Test *n*	ROC-AUC	Balanced Accuracy	MCC
Primary: inactive < 6.0; active ≥ 7.0	365	365	292	73	0.9453	0.7561	0.5972
Active ≥ 6.5; inactive < 6.0	405	389	313	76	0.9460	0.7071	0.5185
Active ≥ 7.0; inactive < 6.5	384	376	303	73	0.9594	0.8061	0.6758
Single cutoff at 6.5	424	400	324	76	0.9460	0.7643	0.5709
Expanded gray zone, 5.5–7.0	345	345	275	70	0.9223	0.7647	0.6782

**Table 3:** Frozen regression partition and leakage-safe descriptor processing.

Characteristic	Development	Locked Test
Compounds	339	85
Bemis–Murcko scaffolds	114	52
Mean pIC50	6.9666	6.9423
Shared scaffolds	0	0
Candidate scaffold partitions evaluated	2000	2000
Frozen partition seed	20,262,779	20,262,779
Initial Mordred descriptors	1613	1613
Final descriptors from full development fit	583	583 *
Descriptors retained in five CV training folds	591, 589, 580, 570, 572	583 *
Activity-distribution comparison	Two-sample KS test	*p* = 0.9922

* Descriptor selection was performed using development data only; the resulting descriptor set was applied unchanged to the locked test set.

**Table 4:** Regression algorithms have advanced from initial benchmarking to predefined configuration selection.

Model	Initial Mean CV R^2^	Initial Pooled OOF R^2^	Best-Configuration Mean CV R^2^	Best-Configuration Pooled OOF R^2^
Extra Trees	0.4453	0.6054	0.4453	0.6059
XGBoost	0.4409	0.5863	0.4699	0.6111
Gradient Boosting	0.4286	0.5843	**0.4909**	**0.6130**

**Table 5:** Final scaffold-aware regression performance.

Evaluation	R^2^	RMSE	MAE
Five-fold scaffold CV, mean ± SD	0.4909 ± 0.3493	0.8238 ± 0.2160	0.6432 ± 0.2233
Pooled scaffold OOF predictions	0.6130	0.8470	0.6439
Locked scaffold-disjoint test, *n* = 85	**0.7560**	**0.6924**	**0.4965**
Locked-test bootstrap 95% percentile interval, *n* = 5000	0.6454–0.8398	0.5448–0.8289	0.3986–0.6032
Repeated scaffold partitions, *n* = 50, mean ± SD	0.6892 ± 0.1515	0.6959 ± 0.1426	0.5279 ± 0.1224

**Table 6:** Regression Y-randomization results.

Characteristic	Value
Number of Y-randomization permutations	100
Observed scaffold OOF R^2^	0.6130
Mean randomized R^2^	−0.2157
SD of randomized R^2^	0.0688
Median randomized R^2^	−0.2092
Maximum randomized R^2^	−0.0964
Randomized models ≥ observed performance	0/100
Empirical *p*-value	0.0099

**Table 7:** Applicability domain assessment of the locked regression test set.

Characteristic	5-NN Distance AD	PCA-Williams AD
Locked-test compounds	85	85
Domain threshold	Distance = 23.7783	h* = 0.3717
Within-domain compounds	76	77
Outside-domain compounds	9	8
Within-domain proportion	89.4%	90.6%
Outside-domain proportion	10.6%	9.4%

* h denotes the critical leverage threshold used to define the PCA-Williams applicability domain.

**Table 8:** Scaffold-aware classification performance.

Metric	Mean Scaffold CV	Pooled OOF	Locked Test
ROC-AUC	0.9068 ± 0.1277	0.9273	0.9453
PR-AUC	0.9512 ± 0.0786	0.9763	**0.9807**
Balanced accuracy	0.8147	0.8588	**0.7561**
MCC	0.6521	0.6675	**0.5972**

**Table 9:** Summary of SHAP descriptor correlation and ranking stability analyses.

Analysis	Regression	Classification
Highest-ranked descriptor	ABC	SlogP_VSA2
Mean |SHAP| of highest-ranked descriptor	0.3182	0.7003
Development–locked rank ρ	0.9909	0.9921
Top-20 pairs with |r| ≥ 0.80	29	16
Top-20 pairs with |r| ≥ 0.90	7	3
Bootstrap perturbations	100	100
Bootstrap rank ρ, mean ± SD	0.5148 ± 0.0363	0.4252 ± 0.0393
Mean top-10 overlap	55.3%	54.4%
Mean top-20 overlap	54.45%	49.75%

## Data Availability

The data supporting the findings of this study are provided in the Supplementary Materials accompanying this article.

## Supplementary Materials

The following supporting information can be downloaded at: https://www.mdpi.com/article/10.3390/ph19091426/s1.

## Abbreviations

The following abbreviations are used in this manuscript:

ML	Machine learning
QSAR	Quantitative structure–activity relationship
ChEMBL	Chemical database of bioactive molecules with drug-like properties
FXIIa	Activated coagulation factor XII
