# Supplementary material for: Development of an Interpretable QSAR Model for Predicting Coagulation Factor XIIa Inhibitors Using Ensemble Machine Learning
Source: Pharmaceuticals (Basel). 2026 Sep 9;19(9):1426. doi: 10.3390/ph19091426 (PMC13610456; doi:10.3390/ph19091426)
Supplement: Supplementary file 1 [file pharmaceuticals-19-01426-s001.zip › FXIIA_Supplementary_Information.pdf]

# Supplementary Information

## Scaffold-Aware and Interpretable Machine-Learning QSAR Modeling of Human Coagulation Factor XIIa Activity

This Supplementary Information provides methodological details supporting the revised FXIIa QSAR analyses. Compound-level data and machine-readable validation outputs are provided separately in FXIIA_Supplementary_Data.xlsx.

## S1. Detailed data-curation criteria

Bioactivity records were reconstructed from ChEMBL release 37 for CHEMBL2821, formally annotated as "Coagulation factor XII" (SINGLE PROTEIN; Homo sapiens), with revised retrieval on 28 August 2026. The primary modeling population was restricted to exact-relation (=), positive, nanomolar IC50 measurements associated with valid standardized structures and without ChEMBL validity warnings. Assays were required to explicitly refer to FXIIa, Factor XIIa, or activated Factor XII. Ki measurements were not pooled with IC50 values. The final primary regression population comprised 424 compounds and 166 Bemis–Murcko scaffolds.

## S2. Replicate handling and sensitivity analysis

IC50 values were converted to pIC50 as 9 − log10(IC50[nM]). Multiple eligible measurements for the same standardized compound were consolidated using the median pIC50. Compounds were not removed from the primary dataset solely because replicate range exceeded 1.0 pIC50 unit. The primary explicit-FXIIa dataset contained 424 compounds, whereas the replicate-strict sensitivity population contained 422 compounds.

## S3. Scaffold partitioning procedure

Bemis–Murcko scaffolds were generated using RDKit and treated as indivisible groups. For regression, 2000 candidate scaffold-disjoint partitions were assessed using predefined model-independent criteria; no descriptors, predictions, cross-validation scores, or locked-test performance were used for split selection. The frozen regression split (candidate 1952; seed 20262779) contained 339 development compounds from 114 scaffolds and 85 locked-test compounds from 52 scaffolds, with zero shared scaffolds. Classification was partitioned independently after gray-zone exclusion; the frozen split (seed 20261115; candidate 284 among 3000 candidates) contained 292 development compounds from 98 scaffolds and 73 locked-test compounds from 45 scaffolds, again with zero shared scaffolds.

## S4. Descriptor calculation and leakage-safe preprocessing

A total of 1613 registered two-dimensional Mordred descriptors were calculated before data-dependent filtering. Descriptors with >20% missing values were removed, followed by variance $\leq 1\times10^{-12}$ and redundancy filtering at $|r|>0.95$. Remaining missing values were median-imputed and scaling was applied where required. The complete preprocessing sequence was refitted independently within each scaffold-CV training fold. The five regression folds retained 591, 589, 580, 570, and 572 descriptors; fitting to the complete 339-compound development set retained 583 descriptors.

## S5. Regression benchmarking and configuration selection

Nine algorithms were benchmarked under five-fold scaffold-grouped cross-validation: Ridge Regression, Elastic Net, RBF-SVR, k-Nearest Neighbors, Random Forest, Extra Trees, Gradient Boosting, HistGradientBoosting, and XGBoost. Extra Trees, XGBoost, and Gradient Boosting were advanced to predefined configuration

selection. Twelve configurations per model yielded 36 candidate configurations and 180 fold-level fits. The frozen Gradient Boosting regressor used 300 estimators, learning rate 0.03, maximum depth 3, minimum samples split 4, minimum samples leaf 3, subsample 0.9, unrestricted maximum features, and Huber loss.

## S6. Regression uncertainty, repeated scaffold validation, and Y-randomization

Locked-test uncertainty was evaluated with 5000 bootstrap resamples drawn with replacement from the fixed 85 observed/predicted pairs. $R^2$, RMSE, and MAE were recalculated and percentile-based 95% intervals were obtained. Partition dependence was assessed across 50 repeated scaffold-disjoint splits. Regression Y-randomization used 100 response permutations. Empirical p-values were calculated as $p\_emp=[1+\Sigma I(M\_i \geq M\_obs)]/(N+1)$, giving a minimum attainable value of 1/101=0.0099.

## S7. Applicability-domain analysis

Two complementary procedures were used. A standardized-space 5-NN distance criterion used a threshold of 23.7783 and classified 76 of 85 locked-test compounds within domain and 9 outside. A PCA-reduced Williams/leverage analysis retained 41 components explaining approximately 95.05% of variance and used $h^*=0.3717$, classifying 77 compounds within domain and 8 outside. Agreement between methods was approximately 91.8%. Domain membership is interpreted as structural/descriptive support, not proof of prediction correctness.

## S8. Classification endpoint and threshold sensitivity

The primary binary definition used pIC50 ≥ 7.0 as active and pIC50 < 6.0 as inactive, with 6.0 ≤ pIC50 < 7.0 treated as a gray zone. This yielded 365 modeled compounds: 267 active and 98 inactive; 59 gray-zone compounds were excluded. Four alternative definitions were evaluated to assess threshold dependence. To isolate threshold effects from repartitioning effects, the scaffold allocation frozen for the primary classification task was preserved. Compounds newly entering from the original gray zone were included in an alternative performance analysis only when their Bemis–Murcko scaffold already had a development/test assignment in the frozen primary partition. Consequently, the threshold-eligible/analyzed populations were 405/389 for active ≥ 6.5 and inactive < 6.0, 384/376 for active ≥ 7.0 and inactive < 6.5, 424/400 for the single 6.5 cutoff, and 345/345 for the expanded 5.5–7.0 gray-zone definition.

## S9. Structural-similarity audit and classification Y-randomization

Each locked classification compound was compared with the development population using RDKit Morgan fingerprints (radius = 2, 2048 bits) and maximum Tanimoto similarity. Mean maximum similarity was 0.7550 and median 0.7816; 30 of 73 compounds had similarity ≥ 0.80 and one reached 0.90. Classification Y-randomization used 100 label permutations. Observed OOF ROC-AUC was 0.9273 versus randomized 0.4889 ± 0.0497; observed MCC was 0.6675 versus randomized −0.0071 ± 0.0691. Both empirical p-values were 0.0099.

## S10. SHAP interpretation, descriptor correlation, and stability

SHAP was used as model attribution. Among top-20 descriptors, regression contained 29 pairs with $|r| \geq 0.80$ and 7 with $|r| \geq 0.90$; classification contained 16 and 3, respectively. Development-versus-locked rank concordance was high (regression $\rho=0.9909$; classification $\rho=0.9921$), but 100 bootstrap perturbations showed only moderate exact-ranking stability (regression mean $\rho=0.5148 \pm 0.0363$; classification $\rho=0.4252 \pm 0.0393$). SHAP results are therefore not interpreted as causal molecular determinants.

## S11. Software and reproducibility information

Verified versions were Python 3.13.15, NumPy 2.1.3, pandas 2.2.3, SciPy 1.16.3, scikit-learn 1.6.1, RDKit 2026.03.5, SHAP 0.47.2, XGBoost 3.4.1, and joblib 1.5.3. Descriptors were generated using the Mordred framework; its exact package version was not retained and is not retrospectively assigned. The accompanying workbook provides curated compound-level data, scaffold assignments, model configurations, predictions, repeated-scaffold results, Y-randomization outputs, applicability-domain assignments, threshold-sensitivity results, structural-similarity audits, and SHAP-related summaries. Trained model binaries and executable notebooks are not included.

## Machine-readable Supplementary Data

The accompanying FXIIA_Supplementary_Data.xlsx workbook contains 18 worksheets arranged in the same analytical order as the Results section: S1 Curated FXIIa Data; S2 Replicate Audit; S3 Classification Data; S4 Classification Threshold Sensitivity; S5 Regression Scaffold Split; S6 Regression Benchmark; S7 Regression Configuration Selection; S8 Regression Locked-Test Predictions; S9 Repeated Scaffold Validation; S10 Regression Y-Randomization; S11 Regression Applicability Domain; S12 Classification Scaffold Split; S13 Classification Performance; S14 Structural-Similarity Audit; S15 Classification Y-Randomization; S16 Regression SHAP; S17 Classification SHAP; and S18 Software and Reproducibility Parameters. This ordering is intended to provide direct, sequential cross-referencing between the main manuscript and the machine-readable Supplementary Data.
